# Tea polyphenols alleviate high fat and high glucose-induced endothelial hyperpermeability by attenuating ROS production via NADPH oxidase pathway

**DOI:** 10.1186/1756-0500-7-120

**Published:** 2014-03-02

**Authors:** Xuezhi Zuo, Chong Tian, Nana Zhao, Weiye Ren, Yi Meng, Xin Jin, Ying Zhang, Shibin Ding, Chenjiang Ying, Xiaolei Ye

**Affiliations:** 1 School of Environmental Science and Public Health, Wenzhou Medical University, Wenzhou 325035, PR China; 2 Department of Clinical Nutrition, Tongji Hospital, Huazhong University of Science & Technology, Wuhan 430030, PR China; 3 Department of Nutrition and Food Hygiene and MOE Key Laboratory of Environment and Health, School of Public health, Huazhong University of Science & Technology, Wuhan 430030, PR China; 4 School of Nursing, Tongji medical college, Huazhong University of Science & Technology, Wuhan 430030, PR China; 5 Present address: School of Public Health, Wenzhou Medical University, WenZhou 325035, PR China

**Keywords:** Green tea polyphenols, High fat, High glucose, Hyperpermeability, NADPH oxidase

## Abstract

**Background:**

Hyperglycemia-induced endothelial hyperpermeability is crucial to cardiovascular disorders and macro-vascular complications in diabetes mellitus. The objective of this study is to investigate the effects of green tea polyphenols (GTPs) on endothelial hyperpermeability and the role of nicotinamide adenine dinucleotide phosphate (NADPH) pathway.

**Methods:**

Male Wistar rats fed on a high fat diet (HF) were treated with GTPs (0, 0.8, 1.6, 3.2 g/L in drinking water) for 26 weeks. Bovine aortic endothelial cells (BAECs) were treated with high glucose (HG, 33 mmol/L) and GTPs (0.0, 0.4, or 4 μg/mL) for 24 hours *in vitro*. The endothelial permeabilities in rat aorta and monolayer BAECs were measured by Evans blue injection method and efflux of fluorescein isothiocyanate (FITC)-dextran, respectively. The reactive oxygen species (ROS) levels in rat aorta and monolayer BAECs were measured by dihydroethidium (DHE) and 2′, 7′-dichloro-fluorescein diacetate (DCFH-DA) fluorescent probe, respectively. Protein levels of NADPH oxidase subunits were determined by Western-blot.

**Results:**

HF diet-fed increased the endothelial permeability and ROS levels in rat aorta while HG treatments increased the endothelial permeability and ROS levels in cultured BAECs. Co-treatment with GTPs alleviated those changes both *in vivo* and *in vitro*. In *in vitro* studies, GTPs treatments protected against the HG-induced over-expressions of p22^phox^ and p67^phox^. Diphenylene iodonium chloride (DPI), an inhibitor of NADPH oxidase, alleviated the hyperpermeability induced by HG.

**Conclusions:**

GTPs could alleviate endothelial hyperpermeabilities in HF diet-fed rat aorta and in HG treated BAECs. The decrease of ROS production resulting from down-regulation of NADPH oxidase contributed to the alleviation of endothelial hyperpermeability.

## Background

Coronary heart disease is the leading cause of death among population in the world [1]. Although the precise mechanism that leads to cardiovascular disease is not fully understood, endothelial dysfunction plays a vital role [2]. Endothelial hyperpermeability, one of the two major manifestations of endothelial dysfunction [3], is one of the initial responses of endothelial cells to insults such as hyperlipidemia and hyperglycemia [4]. Therefore, alleviation of hyperlipidemia/hyperglycemia-related endothelial hyperpermeability will be helpful to prevent cardiovascular disorders.

Oxidative stress is the most common disturbance resulting from hyperglycemia and high fat diet [5,6]. The generation of reactive oxygen species (ROS), chemically reactive molecules containing oxygen, including superoxide anion (O_2_^•−^), hydroxyl radical (·OH), hydrogen peroxide (H_2_O_2_), and peroxynitrite (ONOO^−^), is of particular importance in the alteration of endothelial permeability [7,8]. In vascular endothelial cells, ROS induction is mainly attributable to the production of superoxide anion (O_2_^·−^) catalyzed by NADPH oxidase [7,9]. NADPH oxidase facilitates the electron transfer from NADH or NADPH to molecular oxygen and generates superoxide anion [10]. NADPH oxidases could be composed of membrane-associated subunits gp91^phox^ (NOX_2_), p22^phox^, and cytosolic subunits p47^phox^, p67^phox^, and rac1, the composition is species and tissue dependent [11]. Activation of NADPH oxidase requires phosphorylation and interaction of subunits. Phosphorylated p47^phox^ with its two SH3 domains unmasked binds to p22^phox^ and p67^phox^[12]. The variability of p22^phox^, which is crucial for superoxide anion production in vascular cells [13], is a cause of variation in ROS production in human [14]. The p67^phox^ is inducible and sensitive to a variety of stimuli [15,16]. Based on these, alleviation of NADPH oxidase activation by regulation of NADPH subunit expressions is highly likely to efficiently ameliorate endothelial ROS production and play a role in alleviation of the hyperpermeability induced by hyperlipidemia/hyperglycemia.

Green tea is a popular commodity in Asia. An inverse association between tea consumption and cardiovascular diseases was reported [17]. Green tea polyphenols (GTPs), which comprise 30–40 percent of extractable solids from dried green tea leaves, exhibit significant antioxidant properties [18]. There are multiple routes of their anti-oxidant properties indicated, including chelating metal ions and scavenging free radicals directly due to its poly-hydoxyl structure [19]. However, given the typical daily intake of GTPs, their free radical scavenging or metal chelating abilities are very limited relative to the endogenous anti-oxidant system of organisms [20,21]. On the other hand, the reaction speed of O_2_^·−^ with internal NO in cells is thousands of times higher than its reaction with GTPs. Furthermore, as hydrophilic agents, GTPs have limited access to intracellular ROS that induces endothelial hyperpermeabiliy. Hence, GTPs are less likely to alleviate acute vascular oxidative stress via direct scavenging of ROS [22]. Our previous study showed that GTPs could reduce angiotensin II induced NADPH oxidase activation followed by a corresponding decrease in levels of superoxide anion [22]. Based on the aforementioned data, we hypothesized that GTPs can attenuate diabetes related endothelial hyperpermeability through down regulating the expression of NADPH oxidase followed by reducing the production of ROS.

In the present study, we investigated the effects of GTPs on the endothelial permeability in aortas of rats fed on a HF diet and in cultured BAECs exposed to HG, and the involvements of NADPH oxidase as well.

## Methods

### Ethics statement

The study was carried out in accordance with the Guide for the Care and Use of Laboratory Animals published by the US National Institutes of Health (NIH Pub. No. 85–23, revised 1996). The protocol was approved by the Committee on the Ethics of Animal Experiments of the Huazhong University of Science and Technology (Permit number: S249).

### Reagents and materials

Green tea polyphenols (70% catechins, 10% flavonols, and 20% polymeric flavonoids) were kindly provided by Unilever Health Institute (Vlaardingen, The Netherland). Goat polyclonal antibody (anti-NADPH oxidase, p22^phox^, and p67^phox^) and rabbit anti-β-actin antibody was purchased from Santa Cruz Biotechnology, Inc. (Santa Cruz, USA). Fluorescein isothiocyanate (FITC)-dextran (40 kDa, anionic) was from Molecular Probes, Inc. (Eugene, USA). Dihydroethidium (DHE) and 2′, 7′-dichloro-fluorescein diacetate (DCFH-DA) fluorescent probes were from Beyotime Institute of Biotechnology (Jiangsu, China). Other reagents used were of the highest grade commercially available.

### Animal treatments

Wistar rats were from B&K Universal Group Limited (Shanghai, China). After one week’s acclimation, thirty male Wistar rats, weighting 40-60 g, divided into 5 groups randomly. Four groups of rats fed on a modified HF diet (60% standard chow, 12% sugar, 12% lard, 8% yolk powder, 6% peanuts powder, and 1% milk powder, w/w). Since the 4th week, GTPs solutions of different concentrations (0, 0.8, 1.6, and 3.2 g/L) were served instead of deionized water. One group fed on standard chow as control.

### Measurement of endothelial permeability in rat aorta

At the end of the 26th week, animals were anaesthetized with intraperitoneal injection of ketamine (100 mg/kg body weight) and xylazine (10 mg/kg). Anaesthetic monitoring such as testing of rear foot reflexes and observation of respiratory pattern throughout the procedure was performed. After anaesthesia, Evans blue dye (3%, 1 mL/kg bw) was injected into the tail vein of the rats, thirty minutes later, the animals were sacrificed. The thoracic part of the aorta was isolated, rinsed in normal saline, and weighed. Thirty milligrams of fresh aorta tissue incubated in 0.9 mL dimethylformamide at 50°C for 24 h was used for analysis. The concentration of Evans blue dye was measured spectrophotometrically at 620 nm and presented as μg Evans blue dye per mg wet tissue.

### Measurement of ROS in rat aorta

The ROS levels were measured *in situ* by DHE fluorescent probe, which reacts with ROS and forms ethidium bromide (ETH) that binds to DNA. Fresh cross-sections (10 μm) of frozen aortic arch were incubated with 5 M DHE (37°C, 15 min) in a humidified chamber, then red fluorescence signal was detected with a fluorescence microscope. ROS level was presented as integrated optical density (IOD) per unit area.

### Endothelial cell culture and treatment

Bovine aortic endothelial cells (BAECs, No. C-003-5C) were purchased from Health Science Research Resources Bank (Osaka, Japan). BAECs maintained at 37°C in 5% CO_2_ in Dulbecco’s Modified Eagle’s Medium (DMEM) containing 10% fetal bovine serum. Cells at passages 3–10 were used in this study. For protein expression analysis, confluent cultures were treated with 0.4 μg/mL or 4.0 μg/mL GTPs along with 33 mmol/L glucose in medium (HG group) for 24 h. Cells cultured in 5.5 mmol/L glucose medium were used as controls.

### Cell permeability assay

A previously described method was used for the cell permeability assay [22]. Briefly, BAECs were seeded in the upper chambers of 0.4 μm polycarbonate Transwell filters of a 24-well filtration microplate (Whatman Inc., Clifton, USA). Upon confluence, three groups of the cells were treated with HG (high-glucose, 33 mmol/L) and GTP (0, 0.4 and 4.0 μg/mL) for 24 h. The fourth group was treated with HG alone for 23 hours followed by one hour co-treatment with DPI (10 μmol/L), an inhibitor of NADPH oxidase. After treatment, medium was replaced with fresh phenol red-free DMEM in the presence of FITC-dextran (1.0 μmol/L). The filtration microplate was removed after 2 h incubation, and fluorescence in the medium of the 24-well feeder tray was evaluated at 494 nm excitation and 521 nm emission.

### Measurement of ROS in BAECs

ROS level was determined using a method previously described [23]. Briefly, 1 × 10^5^ endothelial cells per well were seeded onto a 96-well plate, cultured overnight, then exposed to HG with GTPs (0, 0.4, and 4 μg/mL) for 24 h. After exposure, 10 μmol/L DCFH-DA was added to the culture followed by 30 min incubation in the dark. The fluorescence intensity was measured with excitation wavelength at 488 nm and emission wavelength at 525 nm.

### Electrophoresis and immunoblotting

Whole cell extracts were prepared by lysing these cells in extraction buffer (containing 50 mmol/L Tris/HCl, pH 8.0, 150 mmol/L NaCl, 1% Nonidet-P40, 1% sodium deoxycholate, 0.1% SDS, 0.1 mmol/L DTT, 0.05 mmol/L PMSF, 0.002 mg/mL aprotinin, 0.002 mg/mL leupeptin, and 1 mmol/L NaVO_3_). The protein concentration was quantified with BIO-RAD Dc protein assay reagent (Bio-Rad, Hercules, USA). Sodium dodecyl sulfate-polyacrylamide gel electrophoresis (SDS-PAGE) and immunological blotting were performed according to the method provided by Amersham Biosciences. Immunoreactive bands were detected by an ECL plus Western Blotting Detection System (Amersham Biosciences, Little Chalford, UK) according to manufacturer’s instructions. Protein expression was visualized with a chemiluminescent detection system (Syngen, Cambridge, UK) and analyzed by Gel Pro3.0 software (Biometra, Goettingen, Germany).

### Statistical analysis

Quantitative values were expressed as mean ± SEM and the data were analyzed using one-way ANOVA followed by the *Bonferroni* post hoc test at *α* = 0.05. For the statistical analysis, SPSS 14.0 was used.

## Results

### GTPs treatment decreased the endothelial permeability in rat aorta

**Figure 1:**
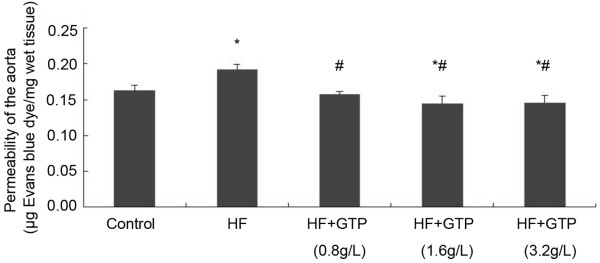
**Effects of GTPs treatment on HF-induced hyperpermeability in rat aorta. Data are expressed as mean ± SEM (N = 6).** * indicates *P* < 0.05 compared to the control and # indicates *P* < 0.05 compared to the HF group.

The endothelial permeability in rat aorta is presented as μg Evans blue per mg wet tissue. As shown in Figure 1, Additional file 1, the Evans blue dye that passed though the endothelium into the vessel wall in the HF group is significantly more than the control group (*P* < 0.05). The GTPs treatment significantly decreased the endothelial permeability in HF fed rats (*P* < 0.05 in comparison with the HF group). In two of the GTPs treated groups (1.6 g/L and 3.2 g/L), the Evans blue dye that passed through the endothelium is even less compared to the control group (*P* < 0.05).

### GTPs reduced ROS production induced by HF in rat aorta and by HG in BAECs

**Figure 2:**
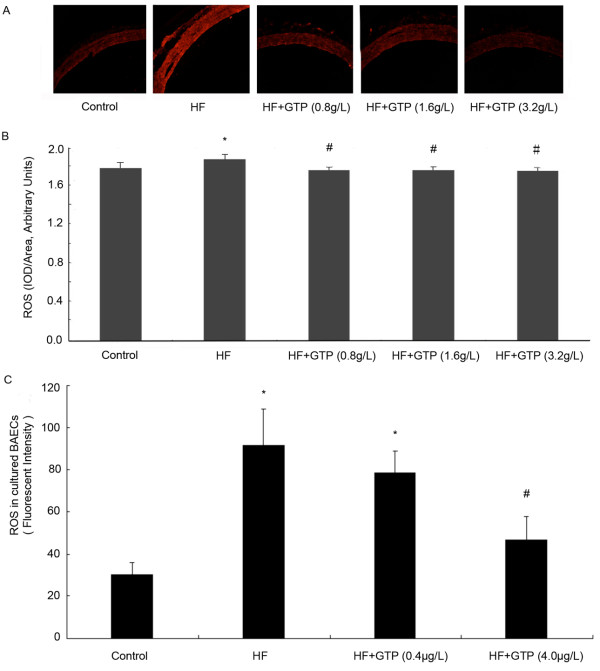
**Effects of GTPs treatment on ROS production in rat aorta and BAECs.** The ROS level in rat aorta was detected by DHE fluorescent probe. As shown in **A**, the fluorescent image of aorta slices were visualized with fluorescence microscope (×200). The fluorescence intensities were measured with IPP image software in randomly selected areas of images captured. The result is shown in **B**. (n = 6, *P < 0.05 vs. control; #P < 0.05 vs. HF). **C**, shows the ROS level in BAECs, the ROS level in BAECs were detected by DCFH-DA fluorescent probe and quantified with spectrometry (excitation at 488 nm and emission at 525 nm). Results represented mean ± SEM. (n = 6, *P < 0.05 vs. control; #*P* < 0.05 vs. HG).

As shown in Figure 2A and B, Additional file 2, HF treatment significantly increased the ROS level of rat aortas compared to the control group. GTPs treatment decreased the ROS level in HF group to control level (*P* < 0.05, GTPs group v.s. HF group). To test whether GTPs decrease the ROS production in cultured endothelial cells, we treated BAECs with 33 mmol/L high glucose and 0.4 or 4.0 μg/mL GTPs for 24 h. As shown in Figure 2C, high-glucose treatment significantly increased the ROS level in BAECs compared to that of BAECs in 5.5 mmol/L glucose medium (*P* < 0.05). Both 0.4 and 4.0 μg/mL GTPs treatments decreased the ROS level induced by HG in BAECs. The ROS level in 4.0 μg/mL GTPs treated cells decreased to 48.9% of that in HG treated cells (*P* < 0.05).

### GTPs down-regulated the elevated expressions of NADPH oxidase subunits (p22^phox^ and p67^phox^) induced by HG

**Figure 3:**
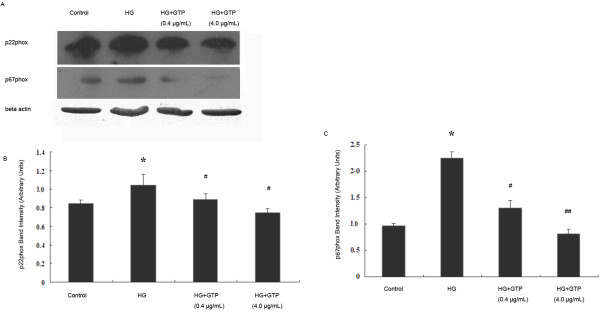
**Effects of GTPs treatment on expressions of NADPH oxidase subunits p22**^**phox **^**and p67**^**phox **^**in BAECs.** Protein levels were determined by Western blotting, and a representative study is shown **(A)**. The bar graph represents three combined experiments. The results of the densitometric analysis (mean ± SEM, N = 3) of immunoblots of NADPH oxidase subunit p22^phox^**(B)**, p67^phox^**(C)**. * indicates *P* < 0.05 compared to the control, # indicates *P* < 0.05 compared to the HG group, and ## indicates *P* < 0.01 compared to the HG group.

To further explore the effects of GTPs on superoxide anion production, the protein expressions of NADPH oxidase subunits p22^phox^ and p67^phox^ were examined (Figure 3A, B, and C). HG significantly increased the protein expression of p22^phox^, and GTPs treatments (0.4 μg/mL and 4 μg/mL) reduced the p22^phox^ expression (*P* < 0.05, Figure 3B). Meanwhile HG increased the protein expression of p67^phox^ more substantially than the expression of p22^phox^. Similarly, GTPs treatments significantly decreased the protein expressions compared with HG group (GTPs (0.4 μg/mL) vs HG *P* < 0.05, and GTPs (4.0 μg/mL) vs HG *P* < 0.01). The results indicated that GTPs down-regulated the increased protein expression of NADPH oxidase resulting in the reduction of superoxide anion production.

### Both GTPs and DPI alleviated the endothelial hyperpermeability induced by HG in cultured BAECs

**Figure 4:**
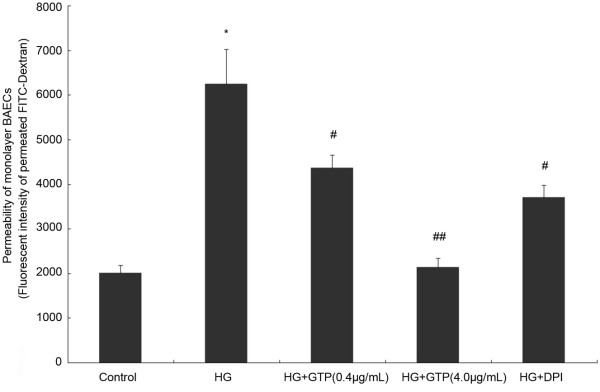
**Effects of GTPs treatment and DPI on HG-induced hyperpermeability in BAECs.** Fluorescence intensity of FITC-dextran that passes through the confluent cell layer to the lower chamber was used to demonstrate the permeability. Data are expressed as mean ± SEM (N = 6). * indicates *P* < 0.05 compared to the control, # indicates *P* < 0.05 compared to the HG group, and ## indicates *P* < 0.01 compared to the HG group.

Whether GTPs can alleviate endothelial hypermeability induced by HG *in vitro* was tested. As shown in Figure 4, similar to the results in the *in vivo* study*,* high glucose induced a significant increase in permeability from 2009 ± 178 fluorescence intensity (FI) in the control group to 6241 ± 783 FI. The permeability was significantly reduced to 4370 ± 278 FI in the cells co-treated with GTPs (0.4 μg/mL) and to 2136 ± 206 FI by GTPs (4.0 μg/mL) co-treatment. Co-treatment with DPI (10 μmol/L 1 h), an inhibitor of NADPH oxidase, also alleviated the endothelial hyperpermeability induced by HG, significantly (*P* < 0.05).

## Discussion

The present study investigated the effects of GTPs on endothelial hyperpermeability, an initial event in the pathogenesis of cardiovascular diseases, induced either by HF *in vivo* or by HG *in vitro*. Major findings of the study are as follows: a. GTPs down-regulated HF-induced hyperpermeability and ROS formation in rat aorta; b. GTPs alleviated HG-induced over-expressions of NADPH oxidase subunits p22^phox^ and p67^phox^, and decreased HG-induced ROS formation in BAECs; c. Co-treatments with GTPs or DPI (inhibitor of NADPH oxidase) ameliorated HG-induced hyperpermeability in BAECs.

Endothelial hyperpermeability is an initial pathological change of cardiovascular diseases. There is little agreement as to what is meant by the term “endothelial permeability” or “vascular permeability”, and there is no consensus about how permeability should be measured [24]. Vascular biologists usually measured the net amount of a solute, typically a macromolecule like plasma albumin, which has crossed a vascular bed and accumulated in the interstitium. However, the movement of macromolecules through the endothelial layer to the sub-endothelial space is of special importance to the development of atherosclerosis and other vascular damages. In the present study, Evans blue, which binds to plasma albumin rapidly *in vivo*, was injected into the vessels of rats, and the dye that pass through the endothelium and trapped in the arterial wall were measured. The result showed that increased blue dye that pass through the endothelium and trapped in the wall of aorta was detected by HF diet, which indicated that the more macromolecules were transported to the sub-endothelial place. GTPs treatment alleviated this endothelial hyperpermeability induced by HF. High fat intake could induce metabolic disorders such as up-regulation of blood glucose and dysregulation of blood lipids in *in vivo* experiments, and the change in blood glucose is much more notable than the change in blood lipids [25]. Hyperglycemia was reported to increase the permeability of micro-vessels in human [26] and high glucose incubation increased the permeability of cultured bovine aortic endothelial cells [27]. Hence, high glucose cultured BAECs were used to explore the potential mechanism of the protective effect of GTPs on endothelial hyperpermeability in the present study. High glucose incubation increased the endothelial hyperpermeability and GTPs treatment alleviated the endothelial hyperpermeability induced by high glucose incubation. These results suggested GTPs intake could potentially be an effective approach for prevention of endothelial dysfunction induced by stressors.

High fat diet may induce vascular endothelial damages through various pathways including increase of ox-LDL levels, increase of advanced glycation end products (AGE), and oxidative stress [6]. ROS production is the vital contributor to the altered permeability in endothelial cells [28]. One possible pathway is that ROS are involved in vascular endothelial growth factor (VEGF) receptor type 2 mediated signaling [29]. While VEGF, formerly named vascular permeability factor, play critical roles in vascular physiology and pathophysiology. Both HF and HG exposure resulted in enhanced ROS production, which is consistent with another study [26], and the increase in ROS production was also alleviated by GTPs treatment. The up-regulations of NADPH oxidase subunits were associated with increased ROS production induced by angiotensin II in ECs [22] and tumor necrosis factor-alpha in human aortic smooth muscle and embryonic kidney cells [30]. A number of studies reported that the expressions and activities of NADPH oxidase are elevated in cardiovascular disorders [9,31]. The high glucose incubation increased the expression of NADPH subunits p22^phox^ and p67^phox^ in BAECs, and similar results were documented in microvascular endothelial cells treated with high glucose [32]. The increased expressions of p22^phox^ and p67^phox^ induced by high glucose incubation were down-regulated by GTPs treatment. Meanwhile, DPI, the most commonly used NADPH oxidase inhibitor, diminished the high glucose induced endothelial hyperpermeability. DPI can abrogate the transport of electrons from reduced flavin, inhibiting the activity of flavin-containing oxidases including NADPH oxidase [33]. These results indicated that GTPs could alleviate the increased NADPH oxidase expression which results in increased ROS production, whereas the increase of ROS production is largely responssible for the high glucose induced endothelial hyperpermeability. Therefore, GTPs alleviated the endothelial hyperpermeability by inhibiting NADPH oxidase.

## Conclusions

The study suggested that GTPs could alleviate the increased NADPH oxidase expression, the elevated ROS production, and the hyperpermeabilities induced by HF in rat aorta and by HG in BAECs. The alleviation of endothelial hyperpermeability was most likely attributable to the down-regulation of NADPH oxidase followed by the corresponding reduction of ROS production.

## Competing interests

The authors declare that they have no competing interests.

## Authors’ contributions

Conceived and designed the experiments: CT XZ CY XY. Performed the experiments: CT XZ NZ WR YM XJ YZ SD. Analyzed the data: CT XY XZ. Contributed reagents/materials/analysis tools: XY CY. Wrote the paper: CT CY XY. All authors read and approved the final manuscript.

## Supplementary Material

Additional file 1: Table S1 Effects of GTPs treatment on HF-induced hyperpermeability in rat aorta. Click here for file

Additional file 2: Table S2 Effects of GTPs treatment on ROS production in rat aorta. Click here for file
